# Contrasting Near-Surface Ozone Pollution in Wet and Dry Year over China

**DOI:** 10.3390/ijerph20020998

**Published:** 2023-01-05

**Authors:** Shuo Ding, Xiaotong Jiang, Changhao Wu

**Affiliations:** Department of Atmospheric Sciences, School of Earth Sciences, Zhejiang University, Hangzhou 310027, China

**Keywords:** ozone, air quality, climate anomalies, drought, health outcomes

## Abstract

The near-surface ozone concentration was evaluated in two typical years with contrasting climatic impacts over the China region induced by El Niño–Southern Oscillation, which had either dry conditions (drought) with intense solar radiation and higher temperatures or wet conditions with opposite meteorological conditions. Surface ozone was observed to aggravate notably by 30% over Northern China in summer and by 50% over Eastern China in autumn in the dry year compared to the wet year. The ozone aggravation was found to be mainly ascribed to the reduced precipitation (relative humidity), enhanced solar radiation and increased temperature rather than primary emission (indicated by carbon monoxide). The health impacts showed the mortality attributable to ozone sharply increased by ~55% in Guangdong while the number of cases dying from ozone-related respiratory diseases per 100,000 population at risk was elevated by ~41% and ~17% for Guangdong (in the Pearl River Delta) and Jiangsu (in the Yangtze River Delta) province (two regions that have been reported to be highly influenced by surface ozone in China), respectively, in the dry year relative to the wet year, indicative of the significant adverse health effects of ozone aggravation. These results highlight the essential contribution of climate anomalies to surface ozone pollution. Efforts to suppress ozone aggravation can be beneficial to public health if extreme drought is predicted, and reasonable policy is implemented.

## 1. Introduction

Tropospheric ozone (O_3_) as an atmospheric pollutant can not only affect atmospheric oxidation capacity but alter the Earth’s radiation budget as well due to its greenhouse effect [1]. Additionally, ozone is toxic to human health [2] and can cause respiratory diseases [3,4]. For this reason, it has been identified as a level-3 risk for human health by the Institute for Health Metrics and Evaluation. Near-surface ozone is a secondary production by photochemical reactions, in which nitrogen oxides (NO_x_) and non-methane volatile organic compounds (NMVOCs) take part [1,5]. It has been reported that daily maximum 8 h average (MDA8) O_3_ presented an increasing trend at a rate of 5% per year in China during the warmer season in the most recent decade, faster than other regions in the world [5,6]. Therefore, it is necessary to probe the factors modulating the variation of ozone concentration in China.

It has been widely known that anthropogenic emissions of precursors of ozone [7] and different meteorological conditions [8,9] can both dictate the surface concentration of ozone. For individual meteorological factors, high temperatures with intense solar radiation can facilitate the photochemical reactions as well as the emission of natural precursors of the ozone [10], rendering ozone pollution severer in the summertime. Clouds can reflect solar radiation thus reducing sunlight received by the surface leading to less ozone formation [11]. Relative humidity (RH) has been revealed to also play a role in the biological behaviors of plants as trees have strong uptake of O_3_ under high RH atmospheric environment [12]. Winds can change the air stagnant condition and transport ozone to downwind regions directly affecting O_3_ concentration [13,14]. Furthermore, based on simulation methods, climatic anomalies induced by El Niño–Southern Oscillation (ENSO) has been reported to influence global ozone distribution as well from atmospheric circulation and weather condition aspects. For example, Xu [15] reported that surface ozone concentration increased in La Niña years while it decreased in El Niño years over the United States during 1993–2013 due to ENSO-induced changes in chemical processes and dynamic transport. With the GEOS-Chem model, Yang [16] showed that summertime surface ozone concentrations in China positively correlated with the ENSO index during 1990–2019 and increased by 20% in Southern China in El Niño relative to La Niña.

Although it has been realized that ENSO could exert influence on tropospheric ozone concentration from model simulation, few studies have contrasted surface ozone pollution in years with distinct extreme climates from an observation perspective in China, such as ENSO-induced extreme precipitation or drought. Years with these two contrasting climatic impacts are expected to have different surface ozone pollution characteristics, which are of crucial significance for governments or policy-makers to take appropriate measures in terms of surface ozone mitigation. In 2016, extreme precipitation events occurred in China with the annual average precipitation ranked first since 1951 [17,18]; thus, this year is defined as a wet year; meanwhile, in 2019, extreme drought happened [19,20] and precipitation was observed to be sharply decreased, thus termed a dry year. Therefore, in this study, based on in situ observations from the China National Environmental Monitoring Center network we investigated the surface ozone pollution in the wet year (2016) and in the dry year (2019) due to ENSO and aims to investigate the linkage between surface ozone pollution and climatic anomalies; meanwhile the health outcomes of ozone pollution were also investigated between both years.

## 2. Materials and Methods

Since 2013, the Ministry of Ecology and Environment of China has established the China National Environmental Monitoring Center (CNEMC) network and started measuring surface atmospheric pollutants all over the nation, including CO, NO_2_, SO_2_, O_3_, PM_2.5_ and PM_10_. Monitoring stations in 114 cities were built in 2013 and the number of monitoring sites has increased up to 1656 covering 378 cities for now [21]. O_3_ was measured by an ozone analyzer (Model 49i), which utilizes ultraviolet photometric technology to measure the ozone concentrations in the atmosphere. In this study, hourly data of O_3_, PM_2.5_, NO_2_ and CO in 2016 and 2019 were used to discern the impact of a climatic anomaly on the surface ozone over China. The data quality control procedures followed previous studies [21,22]. In brief, zero or negative values were set as missing and values that are extreme outliers were removed; then, repeating values that occurred three or more times in a row were also set as missing apart from the first one; finally, outliers with temporal inconsistency were omitted by the method reported in Wu [22].

Meteorological data over 2016 and 2019 encompassing total precipitation (Precip), surface net solar radiation (SSR), the temperature at 2 m (TEM), relative humidity (RH) and wind speed at 10 m (WS) were extracted corresponding to the geographic coordinates of each monitoring site from ERA5 reanalysis product. ERA5 is the fifth-generation and latest reanalysis provided by European Centre for Medium-Range Weather Forecasts (ECMWF) reanalysis. This product offers consistent global climate and weather reanalysis data from 1950 onwards, with a horizontal resolution of 0.25° and a temporal resolution of 1 h [23].

In the present study, both years were compared in seasons (December–February for winter, March–May for spring, June–August for summer and September–November for autumn). For meteorology, the daily maximum for precipitation while daily means for others were used to compute the seasonal means for individual sites. For pollutants, maximum daily 8 h average (MDA8) ozone and daily means of other pollutants were obtained for computing the seasonal average.

In addition, AirQ+ software v.2.1.1 that was developed by the Word Health Organization (WHO) Regional Office for Europe was used to assist in quantifying the health impacts of ozone. This software can calculate the magnitude of the burden and impacts of air pollution on health in a given population. All calculations in the software are founded on the concentration-response functions established by epidemiological studies that are available until 2013 and their meta-analysis [24]. The long-term effects of ozone on the mortality of respiratory diseases in the population aged 30+ years were assessed in 2016 and 2019 following general recommendations of the “Health risks of air pollution in Europe” (HRAPIE) project for the different pollutant-outcome pairs [25]. To perform the calculation, the sum of ozone means over 35 ppb (or 70 μg/m^3^) (*SOMO*35) should be provided by users and it is defined as:

$$SOMO35_{uncorrected}=\underset{i}{\sum }\max\left\{0,C_{i}-35\;\mathrm{ppb}\right\}$$

where *C_i_* is the MDA8 O_3_ and the summation is yearly, i.e., *i* = 1 to 365. When any daily data is missing, *SOMO*35 has to be corrected by the following equation:

$$SOMO35=SOMO35_{uncorrected}\cdot \frac{N_{total}}{N_{valid}}$$

where *N_total_* is the number of all days and *N_valid_* is the number of days having valid values [26]. The Relative Risk (RR) values are recommended as 1.014 (95% CI = 1.005–1.024) per 10 μg/m^3^ for O_3_. Then, at a given mortality incidence of respiratory diseases (per 100,000) and with the total population aged 30+ years, this software could calculate the attributable proportion, the number of attributable cases and the number of attributable cases per 100,000 population at risk related to the mortality ozone causes. Through this effort, the adverse effect of ozone on health outcomes could be further examined.

## 3. Results and Discussions

### 3.1. Meteorology Contrast between 2016 and 2019

**Figure 1:**
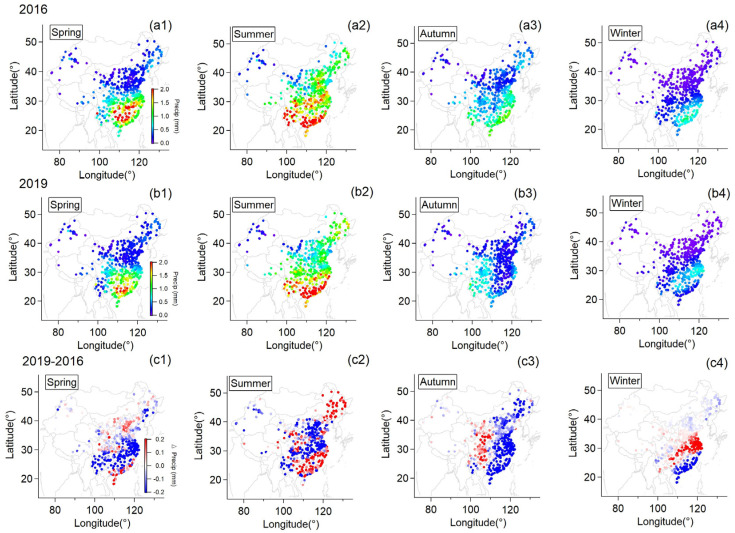
Seasonal mean of daily maximum precipitation for individual monitoring stations for 2016 (**a1**–**a4**) and 2019 (**b1**–**b4**). The difference of this parameter between the two years (**c1**–**c4**).

**Figure 2:**
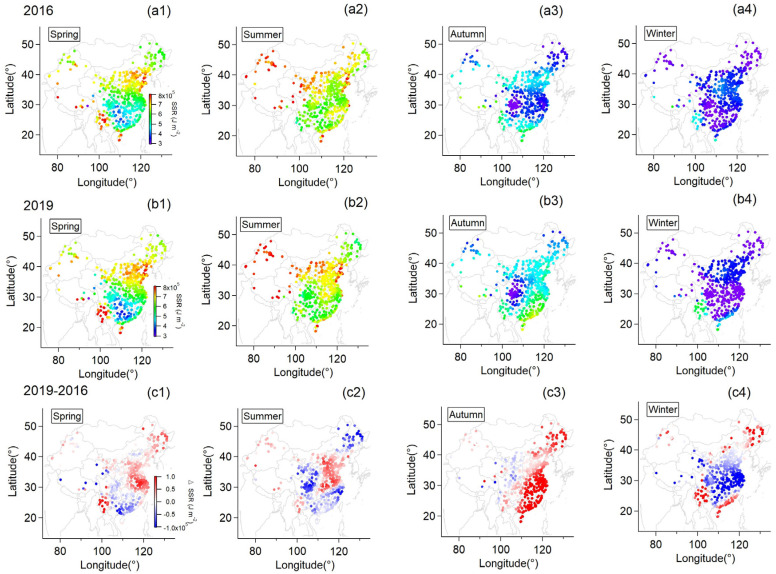
Seasonal mean of daily mean surface net solar radiation (SSR) for individual monitoring stations for 2016 (**a1**–**a4**) and 2019 (**b1**–**b4**); The difference in seasonal averaged daily mean surface net solar radiation between the two years (**c1**–**c4**).

As shown in Figure 1, precipitation had a similar distribution pattern in both spring and summer for both years, except for the fact that higher precipitation was found over Central China in 2016. In this year, the annual average precipitation increased by 16% more than normal level and surpassed the record of 1954 and 1998, ranking the top since 1951, as released by the China Meteorological Administration [17]. It has also been recorded that the May of 2016 was the third wettest May since 1916 over Central Eastern China with extreme precipitation events during this period in the reaches of the Yangtze River [27]. Li [27] reported that the extreme precipitation event could be related to the strong El Niño of 2015–2016. Two ENSO-related processes that were persistent, the western North Pacific anticyclonic anomaly and the southward displacement of the Asian jet stream in the upper troposphere, could both transport more moisture and were considered to explain the extreme precipitation [28]. The most pronounced difference in precipitation was found in autumn whereby the whole of Eastern China was significantly drier in 2019 compared to 2016 with a reduction in precipitation by over 20%. Furthermore, the autumn of 2019 was drier than that of any other year in 2016–2020 (Figure S1). Accompanied by less precipitation, stronger solar radiation enhanced by around 25% was received by the surface in this region (Figure 2(c3)). Therefore, Eastern China had a rather dry autumn in 2019. Xu [19] found that the strong Central Pacific El Niño-related Pacific sea surface temperature anomalies and the super positive Indian Ocean Dipole contributed to 60% and 40% of the drought intensity over the mid-to-lower reaches of the Yangtze River, respectively, where a record-breaking drought was observed since 1979. Similarly, Huo [20] reported the drought event in the autumn of 2019 over Eastern China was strongly influenced by the negative sea-surface temperature anomaly over the southeastern tropical Indian Ocean. In summary, 2016 was rather wet with extreme precipitation events happening this year, while 2019 was a dry year, especially in autumn with prominently lower precipitation and higher net solar radiation due to drought.

### 3.2. The Aggravation of Surface Ozone in the Dry Year

**Figure 3:**
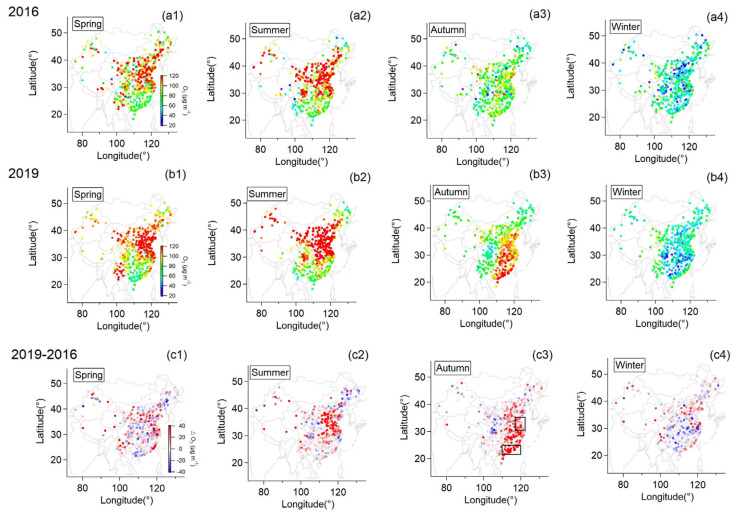
Seasonal mean surface daily maximum 8h average (MDA8) O_3_ at individual monitoring stations for 2016 (**a1**–**a4**) and 2019 (**b1**–**b4**); The difference in surface MDA8 O_3_ between the two years (**c1**–**c4**).

Figure 3 shows the seasonal means of surface MDA8 O_3_ concentration observed by individual monitoring sites in 2016 (a1–a4) and 2019 (b1–b4) while the difference between the two years (2019–2016) is presented in c1–c4. It can be seen that both years had a similar spatial distribution of O_3_ in both spring and summer when Southern China had lower O_3_ concentration than Northern China due to lower primary emission, which suppressed the formation of ozone through secondary reactions [5]. Winter had the least surface ozone compared to other seasons over the nation in both years due to less solar radiation at this time. However, the spatial distribution of surface ozone exhibited pronounced contrast between the autumn of the two years. In 2016, comparable surface ozone concentration was observed over the whole nation but Eastern China had distinctively higher values than Western China by contrast in 2019. This could also be reflected by the difference (ΔO_3_) between years. Compared to 2016, surface ozone increased by about 30% over North China in the summer of 2019, while most of the monitoring stations in eastern China observed a sharp aggravation of surface ozone by over 50% in the autumn of 2019.

**Figure 4:**
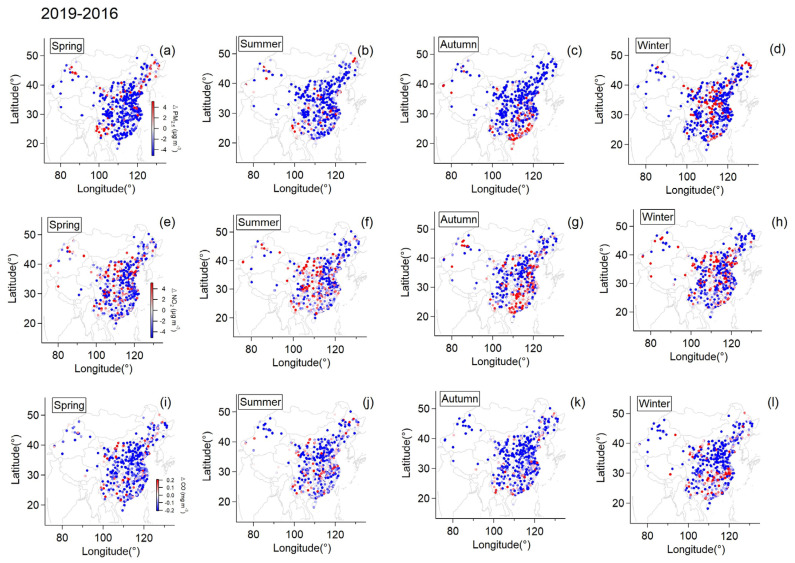
The difference in seasonal averaged daily mean PM_2.5_ concentration (**a**–**d**), seasonal averaged daily mean NO_2_ concentration (**e**–**h**) and seasonal averaged daily mean CO concentration (**i**–**l**) for individual monitoring stations between the two years.

Since anthropogenic emission can have an influence on surface ozone concentration, we further calculated ΔPM_2.5_, ΔNO_2_ and ΔCO between the two years in order to investigate the influence that change in emission may exert on this situation, shown in Figure 4. Owing to the Air Pollution Prevention and Control Action Plan launched by the Chinese government in 2013, all three pollutants were generally reduced in 2019 compared to 2016 for all seasons. However, PM_2.5_ slightly increased in Southern China and Central China in autumn and winter, respectively, while NO_2_ grew by over 2 μg/m^3^ in Eastern and Southern China during the autumn of 2019. Regions that had increased surface ozone concentration partly overlapped where ΔPM_2.5_ and ΔNO_2_ were positive during autumn. Therefore, increasing NO_2_ emission might be a potential factor linked to this case, because NO_2_ serves as one of the crucial reactants for ozone formation [29]. The increased NO_2_ in the autumn of 2019 could provide a reservoir of precursors that was instrumental in the production of ozone; meanwhile, more ozone formation leading to rising secondary formation of PM_2.5_ [30] could give an explanation for the variation of PM_2.5_ during this period.

**Figure 5:**
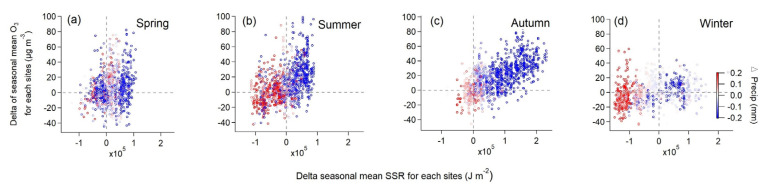
The difference in seasonal mean surface MDA8 O_3_ between the two years as a function of the difference in seasonal averaged daily mean surface net solar radiation (ΔSSR) at individual monitoring stations for all seasons. (**a**) spring, (**b**) summer, (**c**) autumn and (**d**) winter.

Apart from the influence of anthropogenic emissions, climate anomalies could also play a vital role. It clearly shows that ΔPrecip (Figure 1) and ΔSSR (Figure 2) varied similarly to ΔO_3_ (Figure 3) in spatial distribution, particularly over East China in summer and Eastern China in autumn. This relation between ΔPrecip, ΔSSR and ΔO_3_ is further shown in Figure 5. It can be seen that regions with reduced precipitation and increased SSR basically had an increase in surface ozone for summer and autumn. By calculation, SSR increased by 7%, and precipitation was reduced by 32% on average over East China in the summer of the dry year (2019) compared to that of the wet year (2016) when surface ozone concentration increased by about 37% over this region. Meanwhile, this was more pronounced in autumn when precipitation was largely reduced by nearly over 50% with an increase in SSR by 25% over Eastern China and surface ozone was observed to mount by around 40% on average at the same time. Spring and winter were found not to have this clear characteristic as the other seasons. The difference in other meteorological factors between the wet and the dry year was illustrated in Figure S2. ΔRH and ΔPrecip corresponded to each other well in spatial distribution. Positive ΔTem basically coincided with ΔO_3_ at the same area in summer and autumn while negative ΔWS occurred when ΔO_3_ was positive in autumn.

**Figure 6:**
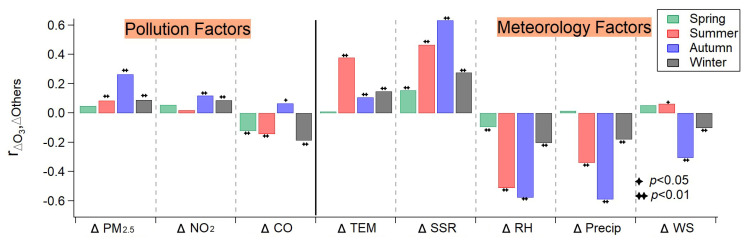
Pearson correlation coefficients of O_3_ difference (ΔO_3_) between the years 2016 and 2019 with that for other pollutants (ΔPM_2.5_, ΔNO_2_ and ΔCO) and meteorological factors (temperature (ΔTEM), surface net solar radiation (ΔSSR), relative humidity (ΔRH), total precipitation (ΔPrecip) and wind speed (ΔWS)) for all seasons. The significance test of passing with *p* < 0.05, *p* < 0.01 or failure is indicated by one star, two stars or no indicator over the top of each bar, respectively.

Figure 6 shows the Pearson correlation coefficients of other pollutants and meteorological factors with ΔO_3_ for all seasons. Higher correlation coefficients were generally found between meteorological factors and ΔO_3_ while a slightly negatively correlation with primary emission (indicated by carbon monoxide) was observed, which suggested the variation in meteorological factors shifted from the wet year to the dry year might be more closely associated with the ozone aggravation than emission. For meteorological factors, ΔO_3_ positively correlated with ΔTEM and ΔSSR while negatively correlated with ΔRH and ΔPrecip (except for spring) in all seasons. However, higher correlation coefficients were only found in summer and autumn. To be specific, ΔO_3_ had a moderate correlation with ΔTEM in summer and with ΔWS in autumn, respectively, while it had a good correlation with ΔSSR, ΔRH and ΔPrecip in both summer and autumn. Consequently, the notable aggravation of surface ozone during summer and autumn in the dry year could be interpreted as follows. In summer, increased surface temperature and decreased precipitation (and RH) resulted in a drier and warmer atmospheric environment. With the contribution of more intense net solar radiation, the formation of ozone could be facilitated. In autumn, higher surface temperature and net solar radiation along with the significantly lowered precipitation brought about persistent drought. When encountering stagnant atmospheric conditions, i.e., lowered wind speed, pollutants were accumulated in the near-surface environment [31]. In this atmospheric condition, it was favorable for surface ozone to be aggravated with the increased NO_2_ emission in the dry year. By using the Kolmogorov–Zurbenko filter, Zhang et al. [32] concluded meteorological factors only had ~20% of the contribution to the long-term trend components of O_3_ in Shenzhen City (in Guangdong province) compared to emission factors. However, in the present study, the influence of climatic anomalies of drought could contribute significantly to ozone variation than is expected in terms of interannual comparisons.

### 3.3. The Health Outcomes of Ozone Aggravation: Case Studies

We further evaluated the health impact of ozone during the wet and the dry year in Jiangsu and Guangdong provinces (two regions having significant ozone aggravation in the dry year and previously reported to be highly influenced by surface ozone in the Pearl River Delta and the Yangtze River Delta [33,34,35,36], marked with black boxes in Figure 3(c3)) as two cases studies; meanwhile, the health outcomes induced by the ozone aggravation between the two years are investigated as well. The indicators as input in the AirQ+ software are listed in Table S1. Note that the mortality incidence (respiratory diseases) was not available for the two provinces, and therefore, we used instead the mortality incidence related to respiratory diseases of the whole nation (provided separately for the urban and rural population) from the China Health Statistics Yearbook [37]. The mortality incidence of the urban and rural populations was integrated into that of the total population based on the respective population proportion. It can be seen that Jiangsu province had higher *SOMO*35 than Guangdong province for both years but Guangdong province was more sensitive to the ozone aggravation with a higher increase in *SOMO*35. For Guangdong province, *SOMO*35 increased by over 50% in the dry year relative to the wet year, which was notably higher than that for Jiangsu province (~27%).

**Figure 7:**
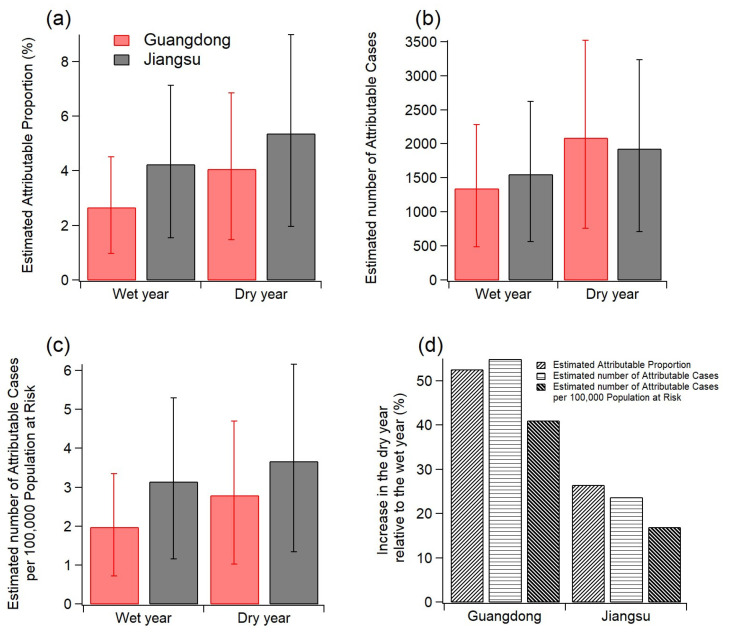
The health outcomes of ozone-related mortality for adults aged 30+: (**a**) attributable proportion due to long-term exposure to O_3_ during the wet and dry years in Guangdong and Jiangsu provinces; (**b**) attributable cases due to long-term exposure to O_3_ during the wet and dry years in Guangdong and Jiangsu provinces; (**c**) the number of attributable cases per 100,000 population at risk due to long-term exposure to O_3_ during the wet and dry years in Guangdong and Jiangsu provinces; (**d**) the relative increase of the three indicators between the wet and the dry years in Guangdong and Jiangsu provinces.

Ozone aggravation could significantly enhance the mortality of respiratory diseases in the population over the age of 30, as illustrated in Figure 7. For Guangdong province, the deaths of respiratory diseases associated with exposure to ozone were 1351 cases, accounting for 2.7% of the total deaths of respiratory diseases in the wet year while the deaths increased sharply by ~55% up to 2094 cases in the dry year. For Jiangsu province, the increase in death cases was less pronounced (~24%) but Jiangsu province had an apparently higher proportion of ozone-related deaths in both wet and dry years compared to Guangdong province. This suggested that although the aggravation of ozone had less influence on the increase in mortality in Jiangsu, ozone contributed more significantly to the mortality associated with respiratory diseases in this region. Furthermore, ozone aggravation escalated the health risk of the population in both provinces as the number of cases dying from ozone-related respiratory diseases per 100,000 population at risk was elevated by ~41% and ~17% for Guangdong and Jiangsu provinces, respectively. Therefore, in general, the aggravation of ozone has a vital influence on the health of the population whereby regions with more intense ozone aggravation had more elevation in the increase in health risk (i.e., Guangdong province) whilst where ozone concentration exceeded WHO air quality standard more was expected to have higher health risk with respect to the mortality induced by exposure to ozone (i.e., Jiangsu province). Since the mortality incidence of the two provinces was not available and the RR value is derived from the single-pollutant analysis of data in 96 metropolitan statistical areas of the United States [2], these results reported here could have some uncertainties. Note that although some factors such as the usage of the mortality incidence of the whole nation rather than that of the two provinces could bring some uncertainties to the results reported here, the consequence that ozone aggravation posed a threat to human health resulting in excess mortality was explicit. In a word, the regular mitigation of ozone pollution together with the suppression of ozone aggravation induced by extreme climate of drought beforehand can be synergistically beneficial to the public health in China.

## 4. Conclusions

Based on the surface ozone data from the China National Environmental Monitoring Center network, we contrasted the ozone pollution characteristics over China in 2016 and 2019. The year of 2016 was considerably wet with abundant precipitation while 2019 was exceptionally dry with extreme drought over the year due to the influence of ENSO. Surface ozone was observed to aggravate notably by 30% over North China in summer and by 50% over Eastern China in autumn during the dry year compared to the wet year. It was found that ΔO_3_ correlated better with ΔPrecip, ΔRH, ΔSSR and ΔTEM in the two seasons but only slightly negatively correlated with primary emissions (indicated by carbon monoxide), suggesting climatic anomalies could play a significant role in the aggravation. The mechanism behind the aggravation in the dry year could be interpreted whereby increased surface temperature and decreased precipitation (and RH) provided a drier and warmer atmospheric environment (drought) where the formation of surface ozone could be substantially facilitated with the contribution of more intense net solar radiation and more NO_2_ supply (especially in the autumn of the dry year).

The health outcomes resulting from the ozone aggravation were also evaluated by performing two case studies in Guangdong and Jiangsu provinces, two of the regions struck by the ozone aggravation. Results from the AirQ+ software showed that deaths from respiratory diseases attributable to ozone had sharply increased by ~55% in Guangdong province while the number of cases dying from ozone-related respiratory diseases per 100,000 population at risk was elevated by ~41% and ~17% for Guangdong and Jiangsu province, respectively, in the dry year relative to the wet year, indicative of the significant adverse effect of ozone aggravation induced by drought on public health.

In summary, climate anomalies can have more of an essential contribution to surface ozone pollution than is expected, i.e., the ozone aggravation sparked by drought, and government and policymakers should not ignore this side effect of drought. The regular control of tropospheric ozone pollution along with the suppression of ozone aggravation can be largely beneficial to public health in the case that extreme drought is predicted, and reasonable policy is implemented. The relationship between extreme weather and ozone pollution is complicated and more work with respect to the contribution of extreme weather to surface ozone pollution is urgently required in the future from numerical methods or model tests. Although further investigations are still needed to better understand the interrelationship between both, this paper aims to make one of the first attempts to link surface ozone to extreme climate/weather over China.

## Data Availability

The data presented in this study are available on request from the corresponding author.

## Supplementary Materials

The following supporting information can be downloaded at: https://www.mdpi.com/article/10.3390/ijerph20020998/s1, Figure S1: Seasonal mean daily maximum precipitation at individual monitoring stations for 2017 (top), 2018 (middle) and 2020 (bottom); Figure S2: The difference in seasonal averaged daily mean temperature (top), relative humidity (middle) and wind speed (bottom) between the two years; Table S1: The parameters used as input in AirQ+ software for Guangdong and Jiangsu province during the wet and the dry year. Reference [37] are cited in the supplementary materials.
