# Supplementary material for: Contrasting Near-Surface Ozone Pollution in Wet and Dry Year over China
Source: Int J Environ Res Public Health. 2023 Jan 5;20(2):998. doi: 10.3390/ijerph20020998 (PMC9859381; doi:10.3390/ijerph20020998)
Supplement: Supplementary file 1 [file ijerph-20-00998-s001.zip › ijerph-2116278-supplementary.pdf]

Supplements

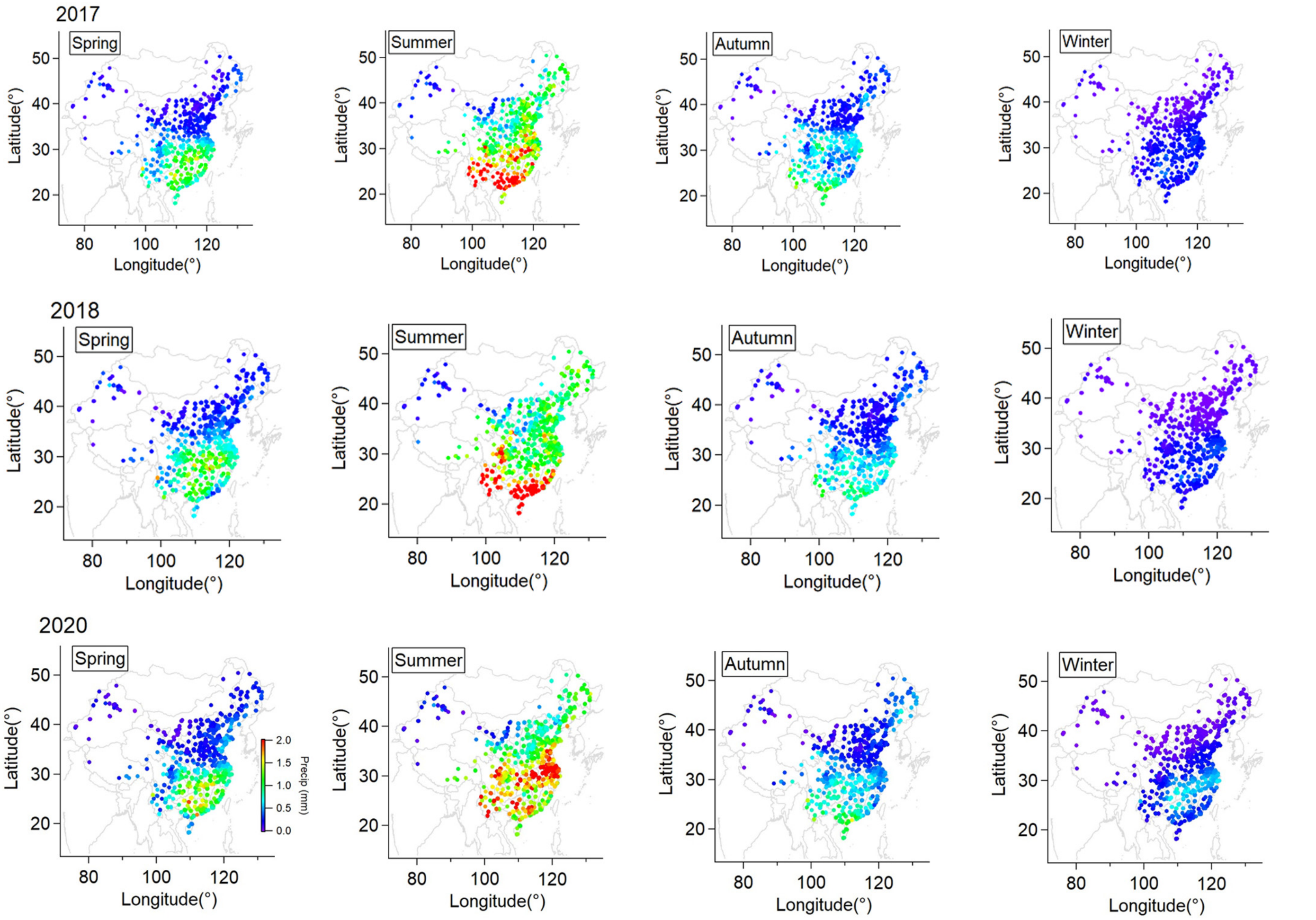

Figure S1. Seasonal mean daily maximum precipitation at individual monitoring stations for 2017 (top), 2018 (middle) and 2020 (bottom).

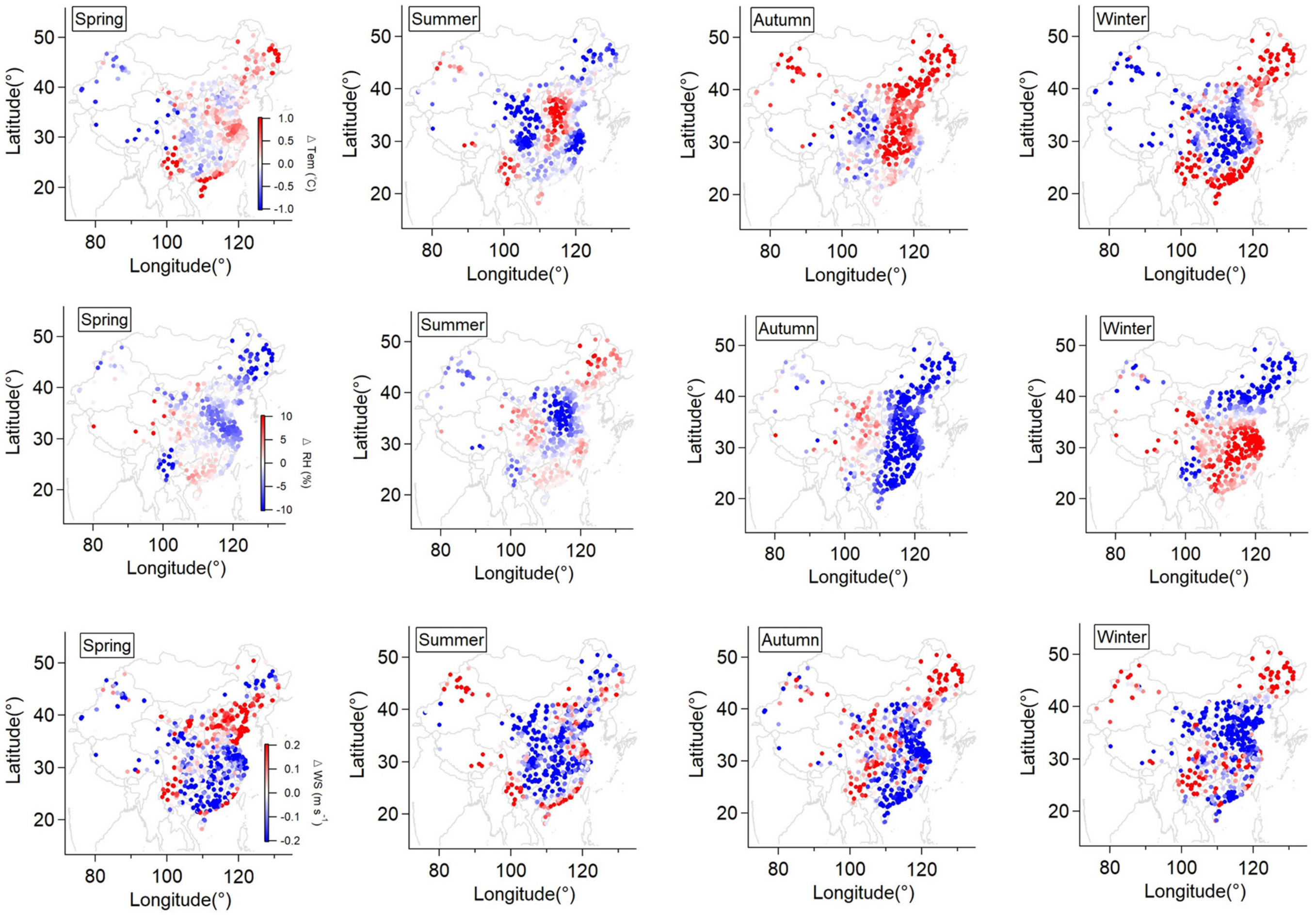

Figure S2. The difference in seasonal averaged daily mean temperature (top), relative humidity (middle) and wind speed (bottom) between the two years

Table S1. The parameters used as input in AirQ+ software for Guangdong and Jiangsu province during the wet and the dry year

Province	2016 (wet year)			2019 (dry year)		
	SOMO35 (μg/m^3)	Total population (Aged 30+) *	Incidence** (/per 100000)	SOMO35 (μg/m^3)	Total population (Aged 30+)	Incidence (/per 100000)
Guangdong	7100.89	68149804	74.25	10924.5	74874979	68.6
Jiangsu	11370.8	49561804	74.25	14479.9	52446930	68.6

* Total population of the two provinces in the two years are from the China Statistical Yearbook (NBSC, 2016 and 2019).

** Incidence denotes the mortality incidence related to respiratory diseases

References

NBSC, N.B.o.S.o.C., 2016 and 2019. China Statistics Yearbook 2016 and 2019. China Statistics Press, Beijing.
